# Using Twitter Data to Monitor Natural Disaster Social Dynamics: A Recurrent Neural Network Approach with Word Embeddings and Kernel Density Estimation

**DOI:** 10.3390/s19071746

**Published:** 2019-04-11

**Authors:** Aldo Hernandez-Suarez, Gabriel Sanchez-Perez, Karina Toscano-Medina, Hector Perez-Meana, Jose Portillo-Portillo, Victor Sanchez, Luis Javier García Villalba

**Affiliations:** 1 Instituto Politecnico Nacional, ESIME Culhuacan, Mexico City 04440, Mexico; ahernandezs1325@alumno.ipn.mx (A.H.-S.); gasanchezp@ipn.mx (G.S.-P.); ltoscano@ipn.mx (K.T.-M.); hmperezm@ipn.mx (H.P.-M.); jportillop@ipn.mx (J.P.-P.); 2 Department of Computer Science, University of Warwick, Coventry CV4 7AL, UK; v.f.Sanchez-Silva@warwick.ac.uk; 3 Group of Analysis, Security and Systems (GASS), Department of Software Engineering and Artificial Intelligence (DISIA), Faculty of Computer Science and Engineering, Office 431, Universidad Complutense de Madrid (UCM), Calle Profesor José García Santesmases, 9, Ciudad Universitaria, 28040 Madrid, Spain

**Keywords:** twitter, data mining, word2vec, CRF, LSTM, geocoding, geoparsing

## Abstract

In recent years, Online Social Networks (OSNs) have received a great deal of attention for their potential use in the spatial and temporal modeling of events owing to the information that can be extracted from these platforms. Within this context, one of the most latent applications is the monitoring of natural disasters. Vital information posted by OSN users can contribute to relief efforts during and after a catastrophe. Although it is possible to retrieve data from OSNs using embedded geographic information provided by GPS systems, this feature is disabled by default in most cases. An alternative solution is to geoparse specific locations using language models based on Named Entity Recognition (NER) techniques. In this work, a sensor that uses Twitter is proposed to monitor natural disasters. The approach is intended to sense data by detecting toponyms (named places written within the text) in tweets with event-related information, e.g., a collapsed building on a specific avenue or the location at which a person was last seen. The proposed approach is carried out by transforming tokenized tweets into word embeddings: a rich linguistic and contextual vector representation of textual corpora. Pre-labeled word embeddings are employed to train a Recurrent Neural Network variant, known as a Bidirectional Long Short-Term Memory (biLSTM) network, that is capable of dealing with sequential data by analyzing information in both directions of a word (past and future entries). Moreover, a Conditional Random Field (CRF) output layer, which aims to maximize the transition from one NER tag to another, is used to increase the classification accuracy. The resulting labeled words are joined to coherently form a toponym, which is geocoded and scored by a Kernel Density Estimation function. At the end of the process, the scored data are presented graphically to depict areas in which the majority of tweets reporting topics related to a natural disaster are concentrated. A case study on Mexico’s 2017 Earthquake is presented, and the data extracted during and after the event are reported.

## 1. Introduction

**Figure 1:**
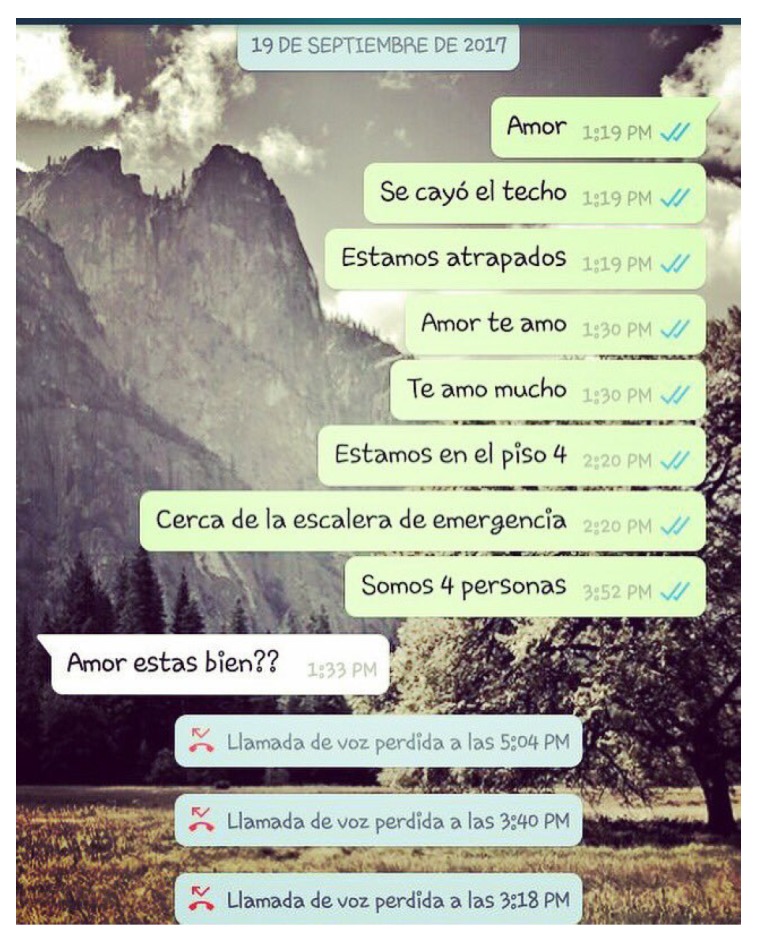
An earthquake survivor uses the WhatsApp messaging system to describe their situation inside a collapsed building. The messages translated to English are *My love. The roof fell. We are trapped. My love I love you. I love you so much. We are on the 4th floor. Near the emergency staircase. There are 4 of us. My love are you ok?* As a result of these messages, rescue teams were able to save the individuals trapped in the rubble [6].

Although state-of-the-art sensors can detect various natural disasters in advance (e.g., Mexico City’s alarm system can timely sense earthquakes originating in the southern states) [1], the devastating consequences of these events in urban areas are usually severe. The relief efforts during and after a disaster are essential for minimizing their negative impact. These efforts are largely the result of motivating the civil society to collaborate with rescue teams, public protection agencies, and security organizations to inform, rescue, and provide restoration. The active participation of civilians in the aftermath not only strengthens the society’s resiliency to a natural disaster but also improves the reliability of the information obtained from non-traditional sources [2,3]. For example, thanks to widespread wireless communication networks and mobile technologies, the dissemination of digital information now serves as a vital way to contact aid services and make appropriate decisions in a fast and more flexible manner [4]. As an example, in the 2010 earthquake in Haiti, the use of instant messages sent by civilians from different locations facilitated the reporting of trapped individuals, the provision of medical assistance, and the delivery of basic needs, such as food, water, and shelter [5]. Personal mobile phones can also be used by survivors to send messages to their relatives and the community at large about their current status, and this information can eventually be forwarded to rescue teams. Figure 1 illustrates an example of an earthquake survivor using their mobile phone to communicate with relatives.

**Figure 2:**
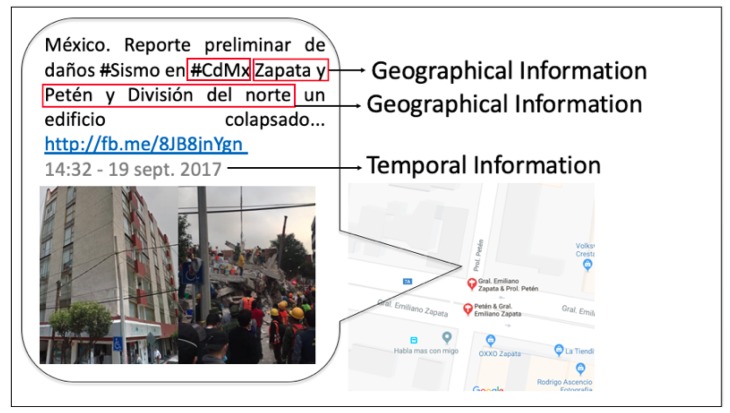
A tweet providing the location (spatial information) of a collapsed building, along with a timestamp (temporal information), one day after the 2017 earthquake in Mexico City. The message translated to English is: *Mexico. Preliminary damage report #Earthquake in #CdMx Zapata and Peten and Division del Norte collapsed building…* It is worth noticing that some users mention places using hashtags. In this example a hashtag #CdMx was used to refer to Mexico City.

Personal mobile devices can be linked to Online Social Networks (OSNs) and enable synchronization among applications, e.g., Twitter, Facebook, and Instagram, which allows users to post and update their activities in real time [7,8]. The creation and prevalence of user-generated content [9] may include temporal and spatial information associated with different events of interest [10]. For the most part, this information is represented by georeferenced patterns that establish a relationship between the posted event and spatiotemporal characteristics of the publishing entity. As an example, an update (tweet) on Twitter that includes temporal and spatial information is shown in Figure 2.

The dynamics of OSN users and their continuous status updates, along with numerous kinds of attachments, such as photos, videos, and documents, can be considered as a social sensor because the data generated on a large scale closely resembles that acquired by traditional sensor systems [11,12]. Below are some characteristics that reinforce the notion that OSNs can be treated as social sensors [13,14]:

- **Sensor operation**: Sensors acquire data from various events as a result of observations. For example, smartphones are equipped with cameras, so users are able to obtain, process, and transmit data in real-time   [12,15].
- **Processing of sensed data**: When the information acquired by traditional sensing systems is processed, geographic information is available if navigation systems, e.g., GPS, are used. Information posted on OSNs may include either specific locations or textual descriptions of a place during an event. Moreover, users can reply, comment, and retransmit an update [16].

Twitter has been popularized for the ease of reading, writing, and collecting data, which are published on a constant basis. Twitter allows users to publish opinions, sentiments, and observations, as well as update their statuses in an asymmetrical form (unlike other OSNs, such as Facebook, a Twitter user’s newsfeed, mentions, and replies remain public by default). Recently, Twitter has been the center of attention in different research fields related to Marketing, Social Sciences, Natural Language Processing (NLP), Opinion Mining, and Predictive analysis [17]. Additionally, several applications are being developed to analyze Twitter data related to daily-life matters. For example, during electoral events, the work in [18] confirmed that a high rate of tweets posted by users shows a correlation with the performance of candidates and the public’s preferences. Event prediction and monitoring can then be carried out by applying connective action theory that links a live event with the reactions of users [19]. For example, it has been demonstrated that events with a negative impact on society can motivate hacker activists to perpetrate cyber attacks [20]. Twitter can then be used as an alternative engine for exchanging information related to natural disasters, such as fires, floods, hurricanes, and earthquakes. Moreover, recent research has demonstrated [21] that Twitter can also be a source of information for spreading awareness of ecological phenomena with well-defined temporal patterns.

In this work, a methodology is proposed that uses Twitter as a social sensor for natural disasters by exploiting the spatial and temporal information associated with the observations and experiences posted by users. The aim of our social sensor is to provide useful geo-temporal patterns that may appear during and after the occurrence of an event, which may be useful to assess the extent of the damages.

By default, tweets are short messages of a maximum of 280 characters in length. Tweets can include well-defined geographic data provided by GPS or manual check-ins. However, it has been reported that only a very small percentage of Twitter users use navigation systems or register places to reference their status [22]. Given this difficulty in determining location, some studies have proposed estimating the location of a tweet by exploiting some of Twitter’s available features, including searching for updates related to certain events within a known geographical region [23], grouping textual patterns associated with user language [24], and parsing Twitter geo-objects to calculate the approximate coordinates from statuses that depict well-defined places [25]. Further, to tackle these limitations, the textual content of a tweet can be examined to determine whether a location is mentioned.

An important contribution of our work is to expand on the idea of examining the textual content of tweets by inspecting the so-called *toponyms* (places implicitly described in a text) from the surge of tweets that emerge during and after a natural disaster. To this end, our proposed approach employs Named Entity Recognition (NER), which is an information extraction method for finding and sorting named entities into pre-defined tags (persons, locations, and organizations) [26,27]. This is achieved by breaking down tweets into word units and classifying them into named entity tags so that a *toponym* can be discovered and geocoded (estimating its spatial information in terms of latitude and longitude coordinates). Detecting places is not a trivial task, and major challenges associated with tweets must be addressed, such as the ungrammatical nature of tweets, as well as informal abbreviations and lexicons (for example, mentioning a location using a hashtag). With respect to temporal information, we cluster values of the time and duration of tweets connected to the event of interest by similarity within a window of time [28]. To capture the semantic, morphological, and contextual richness of each word in a tweet, we perform a word-level analysis by using Word Embeddings [29,30], a widely used algorithm that transforms similar words into a continuous vector space. A sentence-level analysis is subsequently performed to extract semantic and syntactic information from each tweet by employing a Bidirectional Long Short-Term Memory (biLSTM) network [31,32], which is capable of using long-ranged symmetric sequence contexts. After training a Conditional Random Field (CRF) classifier [33] with biLSTM output sequences and their corresponding NER target classes, our methodology predicts locations from tweets. Finally, it applies a Kernel Density Estimation (KDE) algorithm [34] to the classified locations to compute various hotspot heat maps for the event of interest.

We have tested the proposed sensor with (Spanish) tweets from the 2017 Mexico City earthquake. Based on our evaluations, our sensor can accurately capture information that can help authorities, institutions, and volunteers to detect major risk areas and locate missing individuals and shelters.

## 2. Related Work

The detection of events related to natural disasters using OSN data has been the subject of recent research in the fields of sensors, natural language processing, and automatic and statistical learning. The common goal is to detect, monitor, and disseminate information about the event in a timely manner with some degree of trust. As described in [35], Twitter has been recently used as a platform to post diverse information related to various natural disasters, such as wildfires [36], floods [35], hurricanes [37], and earthquakes [38], and it has resulted in situational awareness. Table 1 summarizes the contributions of important works that employed data extracted from Twitter and other OSNs to sense natural disasters.

Although the state of the art provides significant advances in geoparsing *toponyms* from OSNs using NER techniques [39,40,41,42,43,44], some challenges still exist. Important challenges are described below:

- *Vector space feature representations*: a vector space model can capture the relevance of words by assigning them a numerical weight; then, each sentence can be represented as a sparse or dense vector of a vocabulary of size *V*. Some algorithms include One-hot-Encoding, Bag-of-Words, and Tf–IDF (Term frequency–Inverse document frequency) [47]. Such type of codification may fail to preserve semantic, syntactic, and linguistic features, as it cannot establish relationships and similarity patterns among words in a given corpora, making it difficult to examine transitions between contiguous data.
- *Algorithm selection*: Geoparsing techniques based on NER require a suitable algorithm with minor preprocessing to train sequential structures such as tweets; more specifically, long contextual information should be considered in both directions of a word of interest. For this reason, approaches that employ SVM, Feed-Forward Neural Networks, Decision Trees, and single CRF classifiers may be unsuccessful as they assume that words are independent of each other, and rely on previous feature extraction steps. Recent approaches based on feed-forward algorithms for NER classification may have several disadvantages, as tabulated in  Table 2.

In order to clearly define each step of the proposed methodology, the following research questions are raised:

1. How can the semantic, morphological, and linguistic textual patterns that properly represent a word and its surrounding context be preserved?
2. How can a sequential labeling problem such as NER be addressed by capturing contextual information in both directions of a word of interest, and how can it be classified as a *toponym*?
3. Why is it important to scrutinize neighboring named entity tags as state sequences at a sentence level?
4. How should geocoded data and clustered temporal information be statistically scored to depict the dynamics during and after an event?

This work aims to answer these questions. Our main contributions are summarized as follows: (1) A text preprocessing module to remove noisy textual features; (2) Word embedding representations to depict each word of interest, keeping the semantic, syntactic, and linguistic relevance; (3) An NER-based geoparsing strategy (*toponym extractor)* based on a Recurrent Neural Network (RNN) with a CRF output layer to determine the word embeddings that form a tweet and their mapped states (named entity tags); (4) A Geocoder to query Google Maps API with each *toponym*, thus presenting results in latitude and longitude values; (5) A KDE algorithm to graphically depict hotspots from clusters of geocoded *toponyms* in the same spatial area during and after the event of interest.

## 3. Proposed Methodology

**Figure 3:**
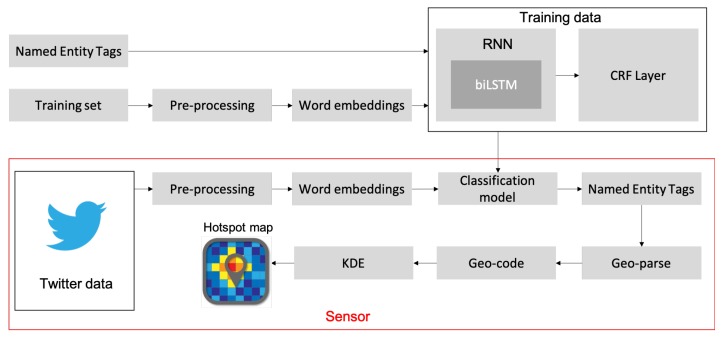
Proposed Twitter-based social sensor for natural disasters.

The block diagram of the proposed sensor is depicted in Figure 3. Each block is briefly described next:

**Training data**

1. *Training set and Named Entity tags*: a training set is prepared with tokenized (segmented text into word units) sentences and manually inspected tweets, along with their corresponding NER tags (Named Entity classes).
2. *Preprocessing*: a step aimed to clean data by removing noisy information, e.g., unnecessary punctuation marks mistakenly added to words, extra spaces, extra line breaks, and bad character encodings, such as emoticons or emojis.
3. *Word embeddings*: Word2Vec [29,30], a well-known word embedding learning algorithm, is used to transform the preprocessed tokens into an *n*-dimensional word vector representation of neighboring context similarity.
4. *biLSTM and CRF*: biLSTM [31,32] is an RNN variation with extended memory capabilities. In this step, word embeddings are used for training by examining words in both directions. This is achieved by adding two separate hidden layers to provide past and future contextual information in specific time frames. Finally, a CRF output layer [33] is used with biLSTM output sequences to exploit their inherent neighboring entity tag transition states over the whole tweet.

**Sensing stage**

1. *Twitter data*: Tweets are scraped using a tool developed in [51]. To be able to filter a meaningful portion of tweets, a compound of queries containing information depicting urban spaces, words, and hashtags related to a natural disaster are stored and grouped into one of the following topics: T∈{disasterareas,missingindividuals,shelters}.
2. *Preprocessing and Word Embeddings*: Tweets scraped in real time are cleaned and transformed into their word embedding representations using the same process as that used in the Training Stage.
3. *Classification model*: This model is obtained druing the training stage and comprises a generalization of word embeddings and entity tags to be used to classify incoming tokenized tweets into named entity tags.
4. *Geoparsing and Geocoding*: Classified tokens are presented as single and sentential words that must be joined correctly to form a *toponym*. A geocoder is developed to resolve *toponyms* to their geographical coordinates by querying Google Maps [52] API and obtaining spatial information in terms of real latitude and longitude values.
5. *KDE*: The occurrence of geocoded *toponyms* in the same spatial region represents the event dynamics as it is a means of understanding what, when, and where users are reporting during and after the event. Such occurrence can be graphically analyzed by using KDE, an algorithm capable of estimating the density of reported locations within some topic ∈T occurring in a well-defined space, such as a hotspot heat map.

### 3.1. Named Entity Tags

Named entities are sequences of words that denote names of things, such as proper names, streets, avenues, and organizations [53,54]. A named entity tag is a discrete class that describes the entity type. Table 3 lists the set of named entities and tags used in the proposed sensor.

### 3.2. Training Set

To build an NER training set, the CoNLL-2002 [55] Spanish dataset was merged with manually inspected tweets using terms in Mexican Spanish related to natural disasters. Tweets were collected by exploting historical messages and hashtags related to Mexico City’s major earthquakes on the following dates: 8 September 2017; 7 June 2014; 17 April 2014; and 20 March 2012. Each training sample comprises a word, $w_{i}$, and its corresponding named entity tag, $y_{i}$. An empty entry in the training set, *X*, represents a sentence boundary. The CoNLL-2002 dataset contains named entity tags with a prefix indicating their position in the sentence, for example, I-LOC indicates that the position is *inside* the sentence and B-LOC indicates that the position is at the beginning of the sentence; thus, we mapped the CoNLL-2002 tags to generic tags, as listed in Table 4.

To illustrate how manually inspected samples (in Spanish) are added to the training set, *X*, Table 5 lists some example tweets whose constituent words are assigned to a named entity tag.

A total of 312,138 different words were used as inputs to a word embedding transform function, as described in Section 3.3.

### 3.3. Word Embeddings

Word-level analysis, also known as word embedding, is a widely used language model transformation [29,30,56,57] whose purpose is to describe words within a certain context. Each word is mapped to a new representation on the basis of its neighboring word co-occurrences in view of semantic, morphological, and linguistic patterns. The main advantage of this kind of language model transformation is its lexical richness, which makes it suitable for handling the non-grammatical nature of data extracted from OSNs; in other vector representation models, this can result in high dimensional data, thus bad weighting factors.

We show the advantages of word embeddings by taking a text describing a location, *Avenida Alvaro Obregon # 286* (a location entity in Mexico City), which can be written in different ways: *Av alv Obregon num 286*, *Ave Alvaro Obregon # 286*, *av. Alvaro Obregon 286*, or *Alv. Obregon 286*. Such variants could be a serious challenge if a feature extraction method that relies on normalizing the frequency of words contained in a document set is employed, e.g., a Vector Representation Model such as the Term frequency–Inverse document frequency (Tf–Idf) algorithm [58]. The generalization employed by such methods may imply a high-dimensional set with a complex interpretation. Instead of using weighting factors, tweets are transformed into vector representations using the Word2Vec-Skip-Gram model [29,30,59]. The Skip-Gram model is widely used for NLP-related tasks by transforming the words composing a sentence into *n*-dimensional vector representations given a desired context, $w_{\psi }$. The model then computes the conditional probability, $p(w_{\psi }|w)$, of a word, *w*, from a given corpus of tweets, *X*. A series of iterations must be performed to tune a parameter β that maximizes the probability over *X*, as formulated in Equation (1):

(1) $$\underset{\beta }{\mathrm{argmax}}\prod_{w_{\in }X}[\prod_{w_{\psi }\in \Psi \left(w\right)}p\left(w_{\psi }|w;\beta \right)],$$

where Ψ(w) is a set of contexts describing a word *w*. To parameterize the Skip-Gram model, it is necessary to make use of the conditional probability $p(w_{\psi }|w;\beta )$ through a Softmax function, as described in Equation (2):

(2) $$p\left(w_{\psi }|w;\beta \right)=\frac{e^{v_{w_{\psi }}\cdot v_{w}}}{\sum e^{v_{w_{\psi }^{\prime }}\cdot v_{w}}},$$

where $v_{w}\in R^{n}$ and ${v_{w}}^{\prime }\in R^{n}$ are the input and output vector representations, respectively, of a word *w*.

### 3.4. Bidirectional Long Short-Term Memory Network

Long Short-Term Memory (LSTM) networks are variants of RNNs and used to solve a wide range of sequential data problems, such as Sentimental Analysis, Speech Recognition, and NER applications [32,60], since they have the ability to capture and exploit historical and long-range dependencies with variable lengths, for example, by capturing past (from the previous words) and future (from the next words) information of a word in a tweet. In text-processing tasks, LSTM networks take words as inputs in a distributed representation of *n*-dimensional vectors with continuous values, in which each word belongs to a finite vocabulary $V\in R^{n\times V}$. In this work, the inputs are the word embedding representations, $v_{w}$, previously transformed by the Skip-Gram model. An LSTM network is constructed with hidden layer updates built into a memory cell, *c*. Each memory block is connected recurrently with an input, forget, and output gate, represented by *i*, *f*, and *o*, respectively. When trained, these gates are able to write, read, and reset information. In Equation (7), each gate is defined:

(3) $$\begin{array}{c} i_{t}=\sigma \left(W_{x^{i}}x_{t}+W_{h^{i}}h_{t-1}+W_{c^{i}}c_{t-1}+W_{0,i}\right), \end{array}$$

(4) $$\begin{array}{c} f_{t}=\sigma \left(W_{x^{f}}x_{t}+W_{h^{f}}h_{t-1}+W_{c^{f}}c_{t-1}+W_{0,f}\right), \end{array}$$

(5) $$\begin{array}{c} c_{t}=f_{t}c_{t-1}+i_{t}\tan h\left(W_{x^{c}}x_{t}+W_{h^{c}}h_{t-1}+W_{0,c}\right), \end{array}$$

(6) $$\begin{array}{c} o_{t}=\sigma \left(W_{x^{o}}x_{t}+W_{h^{o}}h_{t-1}+W_{c^{o}}c_{t-1}+W_{0,o}\right), \end{array}$$

(7) $$\begin{array}{c} h_{t}=o_{t}\tan h\left(c_{t}\right), \end{array}$$

where σ is the sigmoid function; $i_{t}$, $f_{t}$, and $o_{t}$ are the outputs of the input, forget, and output gates, respectively; $c_{t}$ is the output of the cell gate constrained to the size of the hidden vector, $h_{t}$; and *W* and $W_{0}$ are the weights and bias vectors, respectively.

**Figure 4:**
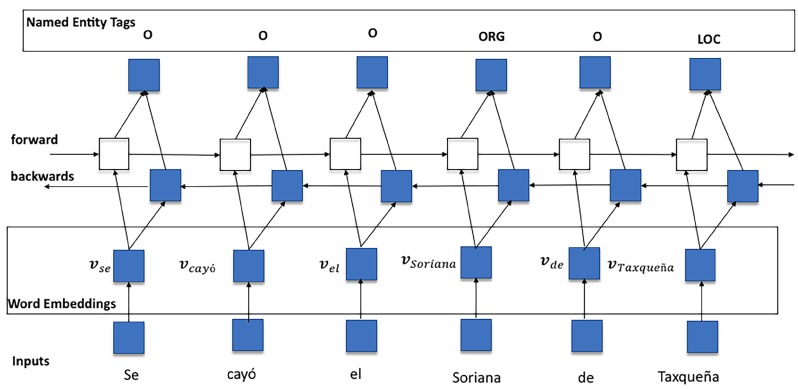
A biLSTM network for NER tasks. English Translation: *Taxqueña’s Soriana has fallen down*.

Although RNNs, including LSTM networks, are useful for working with sequence tagging, they may fail if only past contexts (previous words) are considered. In order to account for the subsequent context, two extra hidden layers are included to process data in a bidirectional fashion. This adaptation is known as a Bidirectional Long Short-Term Memory (biLSTM) network. By training a biLSTM network, the predictive capabilities of a CRF output layer are enhanced by taking advantage of historical information from past vector representations (via forward states) and future vector representations (via backward states). In order to illustrate how a biLSTM works, an example is shown in Figure 4.

### 3.5. Conditional Random Fields

Conditional Random Fields (CFR) [33] are one of the most widely used generative classifiers intended to address NER tasks [61,62,63] as long as their focus is on sequential data. To predict named entity tags, a word-level examination is conducted with a set of sorted and sequential words mapped with an internal state of transitions produced by their corresponding entity tags. When combined with biLSTM networks, the resulting architecture can efficiently process NER sequences with past and future word embedding representations and efficiently predict the entity tag. To this end, a matrix of scores must be computed from the biLSTM outputs, denoted by $f_{\theta }\left({\left[v_{w}\right]}_{1}^{T}\right)$, in which ${\left[v_{w}\right]}_{1}^{T}$ is a sequence of word embeddings associated with a parameter θ, which denotes the score of the *i*-th named entity tag and the *t*-th word embedding. A transition score, ${\left[A\right]}_{i,j}$, is defined to shape the variation from the *i*-th state to the *j*-th state in each pair of consecutive time steps. Lastly, to score a sequence of word embeddings, ${\left[v_{w}\right]}_{1}^{T}$, with a path of tags, ${\left[y_{i,k}\right]}_{1}^{T}$, the sum of the total scores and network scores is calculated according to Equation (8):

(8) $$\sum_{t=1}^{T}\left({\left[A\right]}_{{\left[y_{i}\right]}_{\left[t-1\right]},{\left[y_{i}\right]}_{t}}+{\left[f_{\theta }\right]}_{{\left[y_{i}\right]}_{t},t}\right).$$

Algorithm 1 depicts the steps taken to train the biLSTM-CRF network; *batch* denotes the number of sequences of word embeddings, *epochs* indicates the number of epochs used for training, and ${\left[A\right]}_{i,j},\theta$ are the parameters to update.

**Algorithm 1:** Training Samples.
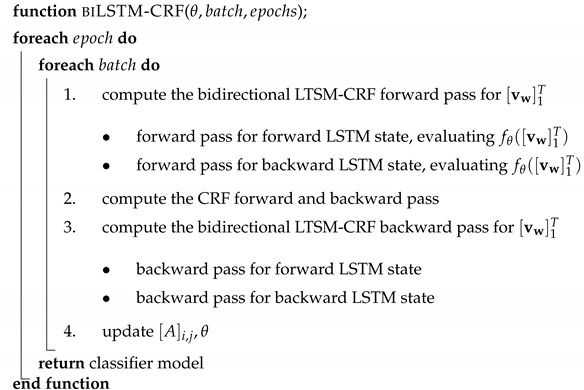


## 4. Sensing Stage

### 4.1. Data Gathering

As presented in [64], it is challenging to retrieve all tweets during and after an event and choose them on the basis of their inherent subjectivity or authenticity of the publishing entity. As concluded in [42], there are two types of queries intended to reduce non-relevant data: (1) keyword-based queries, which search for terms and hashtags determined to be relevant; (2) geographical geo-queries, which search within a bounding box of places of interest. Our proposed sensor monitors hashtags that specifically describe a topic ∈T. We use several geo-queries bounded to the geographical region of interest, e.g., Mexico City. Such geo-queries are aimed to retrieve tweets that contain at least one keyword-based hashtags related to the event of interest, for example for the earthquake that occurred on 19 September 2017 in Mexico City, these keyword-based hashtags are: #sismo, #sismoCDMX, #AyudaCDMX, #FuerzaMexico, #AquiNecesitamos, #derrumbe, #19s, #Voluntarios, #ayudasismoCDMX. For this particular natural disaster, the querying terms are also complemented by well-defined urban spaces from a city [65], e.g., #derrumbe,avenida (which translates into #collapse,avenue), to guarantee that there is, as a minimum, a named place and a particular topic. Twitter characteristics, such as retweets and mentions, contribute to the widespread dissemination of a tweet reporting a location, so these features can be used as a source of temporal information [66]. To exemplify this, a query *q*, related to the tool developed in [20], is shown in Equation (9):

(9) q=[#sismo,ayuda,avenidatlalpan],

where *q* contains the following words in English: #earthqake,help,tlalpan avenue.

### 4.2. Spatial and Temporal Information

**Figure 5:**
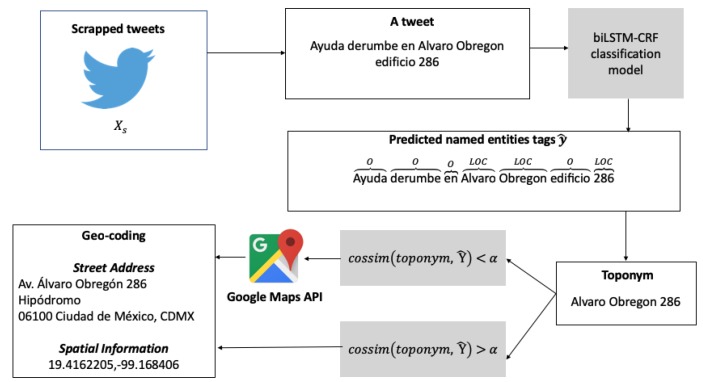
Toponym geocoding.

To be able to sense spatial information, a dataset $X_{s}$ comprising tweets scraped in real time is built for the event of interest. As mentioned before, to classify every tweet into named entities, each tweet must be transformed into its word embedding representation. When several tweets are collected, $X_{s}$ is fed into the classification model to obtain a series of predicted entities $\hat{y}$. Prior to the conversion of predictions into useful *toponyms*, words classified with 0 and PER tags are discarded (their presence is required in the training stage to capture entity tag transitions at the CRF output layer, but they are not needed for *toponym* identification). Furthermore, those classified as LOC and ORG are identified and joined to form a sentence, consequently creating a *toponym*, which is used to request a Google API location. Responses from Google are geocoded in JSON format to form an address with geographic coordinates. To reduce processing times, *toponyms* are appended in a set denoted by $\hat{Y}$ and transformed into a One-Hot-Encoding vector to cluster them using the cosine similarity metric [67] (given some threshold α∈[0,1]). Therefore, if a requested *toponym* is similar to one that is already geocoded, it is assigned the same address and spatial information. Figure 5 depicts the proposed method for geocoding.

To extract temporal information, time windows are employed. In this way, the sensor can grab timestamps, ts, corresponding to the date that a tweet was created. Given the information about when a tweet was initially scraped (the first tweet naming a *toponym)*, its spatial information can be foot-printed. Subsequent retweets (child nodes) originating from an initial tweet are assigned a timestamp equal to the difference between the date of creation of the parent and their current timestamp, i.e., ($ts_{parent}-ts_{children})$, $\left\{\forall ts_{parent}\in {\hat{y}}_{parent},\forall ts_{parent}\in {\hat{y}}_{children}\right\}$. If a *toponym* is identical to others according to the threshold α, the date of creation is then calculated on the basis of those clustered by similarity. Tweets are then sorted by date of creation, from the oldest to the most recent, i.e., $sort({\hat{y}}_{1}\mapsto ts_{1},\ldots ,{\hat{y}}_{n}\mapsto ts_{n})$. For practicality, a 3-day observation window with 7765 unique tweets and 14,155 retweets is applied in our case study.

### 4.3. Kernel Density Estimation

KDE [68] is a statistical method broadly used to graphically visualize hotspots from spatial points distributed on a two-dimensional probability density function [69,70,71,72]. KDE is used on the geocoded *toponyms* to appropriately estimate the distribution of geographic locations within the time windows previously presented in Section 4.2. By plotting with Matplotlib’s Basemap [73], it is possible to visualize geographic areas by topics ∈T, which may include areas likely to be dangerous, plot the zones with the highest rates of missing individuals, and locate aid services via shelters. To quantify the incoming geocoded *toponyms* at a spatial point *g*, Equation (10) is used [72]:

(10) $$f\left(g\right)=\gamma \left(g,h\right)=\frac{1}{Ph}\sum_{\forall \omega_{i}}K\left(\frac{||g-g_{\omega_{i}}{\left|\right|}_{\ell_{2}}}{h}\right),$$

where *h* is the bandwidth; *P* is the total number of pieces of geocoded information of a topic T∈{disasterareas,missingindividuals,shelters} within the time window ω, *i* indexes a single geocoded *toponym* within a time window ω, *K* is is the density function, and $\ell_{2}$ is the vector norm.

## 5. Sensing Information: A Case Study of the 2017 Mexico City Earthquake

**Figure 6:**
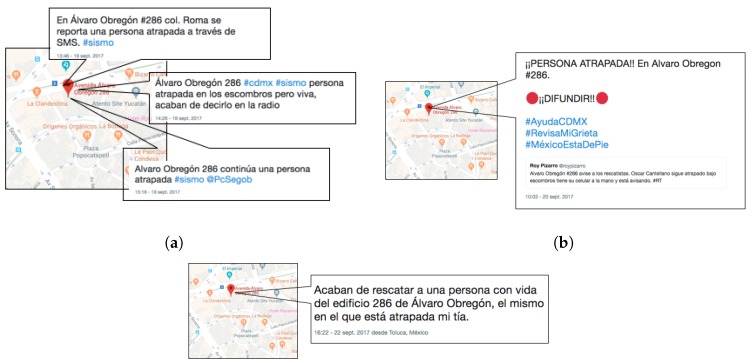
The first report occurs at 1:46 p.m., almost half an hour after the earthquake. The localized entity corresponds to the street *Av. Álvaro Obregón*, number 286, with geographic coordinates 19.4162205, −99.1705947. The other classified entities are similar and ordered temporally until the last report at 4:22 p.m. on the third observation day. (**a**) Users first report that a person is trapped in a collapsed building; (**b**) a day later, users continue reporting that a person is in the rubble, and information is already disseminated in a retweet; (**c**) on the third day, the victim is reported as rescued.

On 19 September 2017 at 1:14 p.m. CST, an earthquake with a 7.1 magnitude on the Richter scale with an epicenter in Axochiapan, Morelos, a state adjacent to Mexico City, impacted the urban infrastructure of the city and surrounding areas. Although the alarm system is efficient when epicenters occur on the Pacific Ocean coast, in the particular case of this natural disaster, the evacuations took place 11 s after the earthquake started because of the lack of sensors near the metropolitan area. It was not to be expected that Twitter users would report information related to the disaster zones. In addition to army and navy personnel, a large number of individuals took to the streets to offer humanitarian aid to people in major risk areas. Days later, a number of official and collaborative shelters were set up in churches, parks, schools, and other places to offer help to the victims. Figure 6 shows a sample of tweets sent over a 3-day observation window.

To compare the proposed sensor with the recent state of the art, a survey was taken of recent works that aimed to address natural disaster monitoring using OSN data with open and available datasets. Table 6 summarizes the works selected to be compared.

In [74], the authors assess the impact of a natural hazard and evaluate different topics: *Caution and advice*, *Displaced people and evacuations*, *Donation needs or offers*, *Infrastructure and utilities damage*, *Injured or dead people*, *Missing*, *trapped or found people*, *Sympathy emotional support*, *Other useful information*, and *Not related or irrelevant*. Then, for each topic, they process tweets by removing noisy patterns, followed by tagging out-of-vocabulary words and normalizing them. Further, they weigh terms using Word2vec and use them for training three classifiers: NB, SVM, and RF. In [76], the authors employ Word Embeddings of a fixed sized and a *simple linear* kernel SVM to classify tweets into one of three topics: *Damage*, *No damage*, and *Not relevant*. To compare these two works with our methodology, datasets provided by [43,74,76] were annotated with the entity classes described in Section 3.1 using Polyglot [78], an NER tagger for multi-lingual purposes, along with other handcrafted rules. Thereafter, to evaluate classification performance, the tagged datasets and *X*, the corpus of tweets used in this work (Mexico City Earthquake), were trained with the pool of algorithms used in [74] and [43,76], as well as with the algorithm (biLSTM-CRF) used in our methodology. For each algorithm, it was assumed that words were preprocessed and transformed into word embedding representations. For biLSTM-CRF, only tags describing a *toponym* (LOC and ORG) are considered. The results are listed in Table 7 in terms of the *precision*, *recall*, and *F-1 score*.

As observed in Table 7, the biLSTM-CRF classifier used to build the proposed sensor performs better on average compared with the the RF, SVM, and NB algorithms. The biLSTM-CRF classifier achieves, on average, a precision = 0.85, a recall = 0.82, and an F1-score = 0.84. Even though word embeddings are used in all approaches, only the biLSTM-CRF classifier can capture the maximum contextual information in both directions of a word embedding and its transitions between NER tags at the state level (sentence), thus improving performance results.

### Visualizing the Social Dynamics via KDE

**Figure 7:**
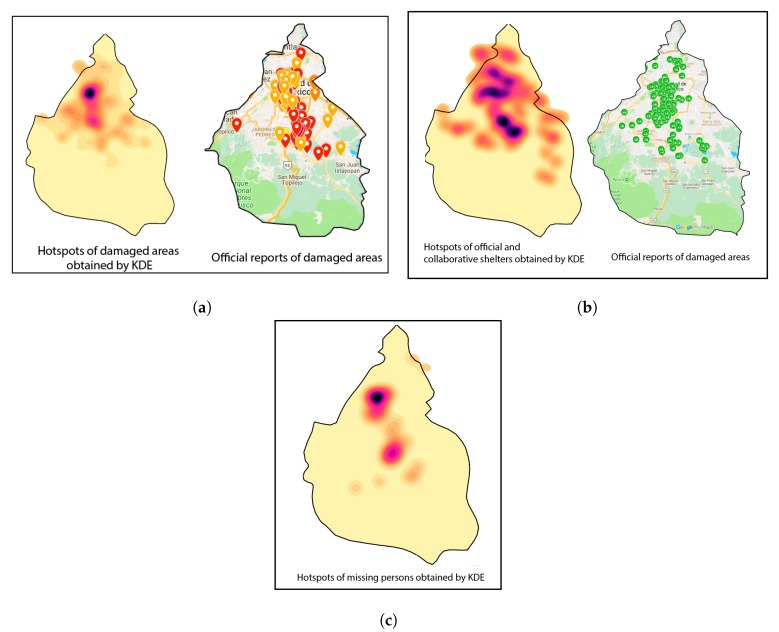
Hotspots maps obtained by applying KDE to the spatial information extracted from data collected over a 3-day window. (**a**) The hotspot map of the estimated spatial locations related to damages and collapses and official reports. (**b**) The hotspot map of estimated spatial locations related to official and collaborative shelters and official reports. (**c**) The hotspot map of estimated spatial locations related to missing persons (there are no official reports of missing persons).

Figure 7a–c depict the hotspots obtained by KDE estimations from the geocoded *toponyms* over a span of three days. These hotspots allow visualizing areas with the highest concentration of tweets reporting a specific topic and naming a *toponym*; i.e., T∈{disasterareas,missingindividuals,shelters}. To validate these results, these hotmaps are compared with two collaborative maps populated with official data verified by Google and the Mexican government (publicly available as *Mapeo Verificado19s* [79]). The information contained in Mapeo Verificado19s’ maps is divided into the following categories:

- **Official Damages**: includes collapsed buildings, major and minor risks, and wall collapses.
- **Official Shelters**: official government assistance and aid.
- **Collaborative Shelters**: non-official collaborative assistance and aid.

In addition, sources of information that contributed in a collaborative way to the population of maps during and after the earthquake are listed below:

- **Mexico City’s Monitor System**: includes major risks, collapsed buildings, and gas hazards.
- **Harvard-Massachusetts Institute of Technology (MIT)**: collaborative data gathered from social media sources.

It is important to emphasize that official and collaborative maps, e.g., *Mapeo Verificado19s*, neither allow for determining the spatial density of the topic of interest nor account for missing persons. This can be a crucial disadvantage in cases where it is necessary to examine the dynamics and evolution of an event of interest on the basis of incoming reports. The authenticity of *toponyms* is tested by searching official addresses published by Mexico’s federal government [80]. This information can be collected only after civil protection units verify the geographical areas of the disaster and issue an official statement. Unfortunately, there were no oficial data for this natural disaster related to aid, shelter, and missing persons. The proposed sensor has then the potential to assist in estimating in real-time the geographical regions with the largest density of tweets associated with a specific topic of interest, enabling information to be disseminated without subjecting responders to the risks associated with on-site verification. Table 8 lists the most common geocoded *toponyms* transformed into Google API addresses found by our sensor. These locations have also been officially declared as disaster areas by Mexico’s federal government.

## 6. Conclusions

In this work, a methodology that uses Twitter as a social sensor is proposed. This is accomplished by employing an information sequential extraction procedure known as Named Entity Recognition (NER), which aims to describe mentioned entities, such as places, persons, and organizations. The methodology considers the semantic, morphological, and contextual information about each word composing a tweet and its surrounding context, thus allowing to properly identify a named place (*toponym*). To achieve this, words are tokenized and transformed into word embeddings to represent them as vectors with rich syntactic and semantic relationships that are established by neighboring words. To ensure that a high classification accuracy of the sequential data is achieved with out heavily relying on handcrafted feature extraction techniques, a Recurrent Neural Network variant, i.e., a Bidirectional Long Short-Term Memory (biLSTM) network, is used. Specifically, the biLSTM network deals with long-distance dependencies, which feed-forward algorithms, such as NB, SVM, and RF, cannot handle. This is achieved by considering contextual information in both directions of a word in a tweet. By using a CRF output layer with the biLSTM network, NER tag transitions over the word embeddings are accounted for.

In the presented case study, geo-queries related to the earthquake of 19 September 2017 in Mexico City were used to retrieve tweets with specific keyword-based hashtags. After classifying Tweets with NER tags and joining them to form useful *toponyms*, these *toponyms* were geocoded in terms of addresses and latitude and longitude coordinates by means of Google’s API. Finally, a KDE algorithm was computed to visualize the spatial density of geocoded *toponyms* from topics related to disaster areas, missing individuals, and shelters. Our results show that addresses and coordinates obtained by our methodology coincide with the ones reported by civil protection units and with official data from Mexico’s federal government. Collaborating with the government and civil organizations to improve the timely detection of disaster areas, finding missing individuals, and locating shelters in real-time by using our proposed methodology is part of our future work. 

## Figures and Tables

**Table 1:** Related work that contributes to natural disaster sensing using data extracted from Twitter and other OSNs.

Title	Description
Earthquake Shakes Twitter Users: Real-time Event Detection by Social Sensors.	To detect a target event, this work classifies tweets on the basis of features such as keywords and the number of words given a context. Then, the methodology estimates a probabilistic spatiotemporal model to find the center and the trajectory of the target event. To this end, each Twitter user is assumed to be a sensor. Then, Kalman particle filtering is applied for location estimation with ubiquitous/pervasive computing. The authors claim that a 96% probability of correctly detecting an earthquake can be achieved by monitoring textual features [39].
Public health implications of social media use during natural disasters, environmental disasters, and other environmental concerns.	This work analyzes how social media can be used to disseminate information, predict data, and provide early warnings within the context of environmental awareness and health promotion. The work also analyzes how social media can be used as an indicator of public participation in environmental issues. The authors found evidence supporting social media as a useful surveillance tool during natural disasters, environmental disasters, and other environmental concerns. The work shows that public health officials can use social media to gain insight into public opinions and perceptions. Moreover, the work shows that social media allows public health workers and emergency responders to act more quickly and efficiently during crises [40].
Real-Time Crisis Mapping of Natural Disasters Using Social Media.	In this work, the authors propose a social media crisis mapping platform for natural disasters that uses statistical analysis with geoparsed real-time tweet data streams matched to locations from gazetteers, street maps, and volunteered geographic information. Geoparsing results are benchmarked against existing published work and evaluated across multilingual datasets. Two case studies are presented to compare five-day tweet crisis maps compiled from verified satellite and aerial imagery sources for official post-event impact assessment by the US National Geospatial Agency [41].
Tweedr: Mining Twitter to Inform Disaster Response.	In this paper, the authors introduce Tweedr, a Twitter-mining tool that extracts actionable information for disaster relief workers during natural disasters. The Tweedr pipeline consists of three main parts: classification, clustering, and extraction. In the classification phase, they use classification methods, namely, Latent Dirichlet Allocation (LDA), Support Vector Machines (SVM), and Logistic Regression, to identify tweets reporting damage or casualties. In the clustering phase, they use filters to merge tweets that are similar. Finally, in the extraction phase, they extract tokens and phrases that report specific information about different classes of infrastructure damage, the types of damage, and casualties [42].
A Linguistically-driven Approach to Cross-event Damage Assessment of Natural Disasters from Social Media Messages.	In this work, the authors focus on the analysis of Italian social media messages for disaster management. Their aim is to detect those messages conveying critical information for the damage assessment task. The main novelty of this study is the focus on out-of-domain and cross-event damage detection and the investigation of the most relevant tweet-derived features for these tasks. They conducted different experiments by resorting to a wide set of linguistic features to qualify the lexical and grammatical structure of a text, as well as ad-hoc features specifically extracted for this task [43].
Combining Machine Learning Topic Models and Spatio-temporal Analysis of Social Media data for Disaster Footprint and Damage Assessment.	The authors propose a crisis mapping system by analyzing the textual content of disaster reports from a twofold perspective. A damage detection component employs an SVM classifier to detect mentions of damage among emergency reports. A novel geoparsing technique is proposed and used to perform message geolocation. They report a case study to show how the information extracted through damage detection and message geolocation can be combined to produce accurate crisis maps. The crisis maps detect both highly and lightly damaged areas, thus opening up the possibility to prioritize rescue efforts where they are most needed [44].
From Social Sensor Data to Collective Human Behaviour Patterns: Analysing and Visualising Spatio-temporal Dynamics in Urban Environments.	This paper presents an approach to analyzing social media posts to assess the footprint of and the damage caused by natural disasters by combining machine learning techniques (LDA) for semantic information extraction with spatial and temporal analysis (local spatial autocorrelation) for hotspot detection. The results demonstrate that earthquake footprints can be reliably and accurately identified. The results also show that a number of relevant semantic topics can be automatically identified without a priori knowledge, revealing clearly differing temporal and spatial signatures. Furthermore, a damage map that indicates where significant losses have occurred is also presented [11].
The Performance of Publicness in Social Media: tracing patterns in tweets after a disaster	The authors propose a computer-assisted discourse analysis—specifically, a corpus-linguistic-informed analysis of half a million tweets—in order to describe four main public discursive moves that were prevalent during the earthquake in Aotearoa, New Zealand, in 2011. The final results describe how people employ their social media communication at critical, reflexive moments, such as in the aftermath of disaster [45].
Spatio-Temporal Distribution of Negative Emotions in New York City After a Natural Disaster as Seen in Social Media	In this paper, the authors propose a sentiment analysis technique termed *Extracting the Meaning Of Terse Information in a Visualization of Emotion (EMOTIVE)*, which uses spatial regimes regression to find significant associations of negative emotional responses by using social media posts over space and time in the aftermath of a natural disaster. The process can be used as a guide to identify those areas and populations in the most need of care [46].

**Table 2:** Disadvantages of algorithms employed for NER classification

Algorithm	Disadvantages for NER Tasks
Decision Trees (DT) and Random Forests (RF)	In [48], the authors conclude that DT and RF can create useful rules for sentence segmentation and partial parsing for NER classification and *toponym* identification, but they do not adequately consider linguistic or semantic knowledge. DT and RF are, unfortunately, prone to overfitting, and their complexity may be exponential in online learning scenarios and for high-dimensional sets, such as textual corpora.
Naive Bayes (NB)	The authors of [49] suggest that NB can be used for nonlinear NER classification by computing the posterior probability of a word associated with a specific tag. Despite this, NB approaches may fail if they do not consider an intermediate representation of the word-by-word composition of each sentence, especially for examining the sequential relationships among the input words.
Support Vector Machines (SVM)	SVM-based applications have been widely used for NER tasks [50] to efficiently increase accuracy scores. Although designed to maximize the decision boundaries for binary classification problems, *kernel* *tricks* can help to adapt nonlinear data to different dimensions, as well as adjust training steps for multi-class datasets. In any case, SVM architectures depend on previous handcrafted feature extraction techniques, which may result into high-dimensional and sparse results, making it time-consuming for sequential problems such as NER.
Single Conditional Random Fields (CRF)	CRF is one of the top-ranked generative algorithms used for NER, as studied in ref. [50]. Its main difference from discrete classification is that data are represented as sequences of tags with mapped classes for each one. Predictions are presented by maximizing the log-likelihood of the state sequences given the observed classes. Indeed, similar to SVM and DT, generative models such as CRF applied on its own cannot properly generalize long-range dependencies, such as the contextual and lexical features needed for NER.

**Table 3:** Entity tags used for classification.

Named Entity Tag Type	Description
LOC	Location representation, e.g., a street, avenue, region, or country
ORG	Reference to an organization, institution, or establishment
PER	Reference to a person or a group of people
O	Any other criteria

**Table 4:** Named entity tags used in the training set.

CoNLL-2002 Tag	Generic Tag
I-LOC, B-LOC	LOC
I-ORG, B-ORG	ORG
I-PER, B-PER	PER
O, I-MISC, B-MISC	O

**Table 5:** Examples of tweets with their corresponding named entity tags.

Tweet in Spanish	English Translation
$\overset{O}{\overset{︷}{\mathrm{ayuda}}}\overset{O}{\overset{︷}{\mathrm{gente}}}\overset{O}{\overset{︷}{\mathrm{atrapada}}}\overset{O}{\overset{︷}{\mathrm{en}}}\overset{LOC}{\overset{︷}{\mathrm{edificio}}}\overset{LOC}{\overset{︷}{\mathrm{Alvaro}}}\overset{LOC}{\overset{︷}{\mathrm{Obregon}}}$	help people trapped in a building located in Alvaro Obregon
$\overset{O}{\overset{︷}{\mathrm{derrumbe}}}\overset{O}{\overset{︷}{\mathrm{en}}}\overset{O}{\overset{︷}{\mathrm{el}}}\overset{LOC}{\overset{︷}{\mathrm{multifamiliar}}}\overset{O}{\overset{︷}{\mathrm{de}}}\overset{LOC}{\overset{︷}{\mathrm{av}.}}\overset{LOC}{\overset{︷}{\mathrm{tlalpan}}}$	a collapsed department building on tlalpan avenue
$\overset{O}{\overset{︷}{\mathrm{se}}}\overset{O}{\overset{︷}{\mathrm{cay}\text{ó}}}\overset{O}{\overset{︷}{\mathrm{el}}}\overset{ORG}{\overset{︷}{\mathrm{Soriana}}}\overset{O}{\overset{︷}{\mathrm{de}}}\overset{LOC}{\overset{︷}{\mathrm{Taxque}\text{ñ}a}}$	Taxqueña’s Soriana has fallen down

**Table 6:** Recent works used to compare the proposed sensor.

Titles	Natural Disaster	Dataset	Algorithms Employed	Algorithm with Overall Best Performance Metric Reported	Year
Twitter as a Lifeline: Human-annotated Twitter Corpora for NLP of Crisis-related [74]	Napa California Earthquake, USA	Publicly available on *Resources for Research on Crisis Informatics* [75]	NB, SVM, and RF with Word Embeddings	NB with 82% accuracy	2016
A linguistically-driven approach to cross-event damage assessment of natural disasters from social media messages [43] A Big Data Crisis Mapping System Based on Damage Detection and Geoparsing [76]	L’Aquila and Emilia earthquakes from 2009 to 2014, Italy	Publicly Available on *Project SoS* [77]	SVM + Word Embeddings, SVM and NLP + POS tags	SVM + Word Embeddings with 88% F1-score	2018

**Table 7:** Results comparison.

Dataset	Classifier	Named Entity Tag	Precision	Recall	F-1 Score
19 September 2017 Mexico Earthquake	biLSTM-CRF	LOC	0.83	0.76	0.80
19 September 2017 Mexico Earthquake	biLSTM-CRF	ORG	0.83	0.86	0.85
2009–2014 L’Aquila and Emilia earthquakes, Italy	biLSTM-CRF	LOC	0.84	0.84	0.84
2009–2014 L’Aquila and Emilia earthquakes, Italy	biLSTM-CRF	ORG	0.79	0.69	0.74
2014 Napa California Earthquake, USA	biLSTM-CRF	LOC	0.93	0.90	0.92
2014 Napa California Earthquake, USA	biLSTM-CRF	ORG	0.88	0.87	0.87
		**Average**	0.85	0.82	0.84
19 September 2017 Mexico Earthquake	RF	LOC	0.89	0.19	0.31
19 September 2017 Mexico Earthquake	RF	ORG	0.89	0.18	0.30
2009–2014 L’Aquila and Emilia earthquakes, Italy	RF	LOC	0.74	0.60	0.66
2009–2014 L’Aquila and Emilia earthquakes, Italy	RF	ORG	0.76	0.29	0.42
2014 Napa California Earthquake, USA	RF	LOC	0.60	0.25	0.35
2014 Napa California Earthquake, USA	RF	ORG	0.75	0.26	0.39
		**Average**	0.77	0.30	0.40
19 September 2017 Mexico Earthquake	SVM	LOC	0.76	0.48	0.59
19 September 2017 Mexico Earthquake	SVM	ORG	0.73	0.78	0.64
2009–2014 L’Aquila and Emilia earthquakes, Italy	SVM	LOC	0.75	0.57	0.65
2009–2014 L’Aquila and Emilia earthquakes, Italy	SVM	ORG	0.82	0.25	0.38
2014 Napa California Earthquake, USA	SVM	LOC	0.63	0.44	0.52
2014 Napa California Earthquake, USA	SVM	ORG	0.82	0.24	0.37
		**Average**	0.75	0.45	0.53
19 September 2017 Mexico Earthquake	NB	LOC	0.88	0.19	0.31
19 September 2017 Mexico Earthquake	NB	ORG	0.86	0.18	0.30
2009–2014 L’Aquila and Emilia earthquakes, Italy	NB	LOC	0.79	0.46	0.58
2009–2014 L’Aquila and Emilia earthquakes, Italy	NB	ORG	0.84	0.24	0.37
2014 Napa California Earthquake, USA	NB	LOC	0.51	0.57	0.54
2014 Napa California Earthquake, USA	NB	ORG	0.78	0.26	0.39
		**Average**	0.70	0.47	0.42

**Table 8:** Geocoded addresses and coordinates found by the sensor and officially declared as disaster areas.

	Geocoded Address	Geocoded Coordinates	Tweets	Retweets
1	Rancho Tamboreo & Calz de las Brujas, Nueva Oriental Coapa, 14300 Ciudad de México, CDMX	19.2965695, −99.1328497	135	368
2	Calz. de Tlalpan 20, Conjunto Urbano Tlalpan, 04400 Ciudad de México, CDMX	19.3385929, −99.1446581	126	331
3	Av. Álvaro Obregón 286 Hipódromo 06100 Ciudad de México, CDMX	19.4162255, −99.170594	112	250
4	Amsterdam 25, Hipódromo, 06100 Ciudad de México, CDMX	19.4158929, −99.1701461	109	204
5	Calle Torreón & Viad. Miguel Alemán, Piedad Narvarte, 06760 Ciudad de México, CDMX	19.4025116, −99.1634792	104	237
6	Edimburgo & Escocia, Col del Valle Centro, 03100 Ciudad de México, CDMX	19.3875319, −99.1656197	103	228
7	Amsterdam & Calle Laredo, Hipódromo, 06100 Ciudad de México, CDMX	19.4129041, −99.1730674	97	143
8	Av. Álvaro Obregón 284, Hipódromo, 06100 Ciudad de México, CDMX	19.4162562, −99.1704433	96	127
9	Coahuila 286 Hipódromo, 06700 Ciudad de México, CDMX	19.410391, −99.1685889	94	164
10	Simón Bolívar 190, Obrera, 06800 Ciudad de México, CDMX	19.4221723, −99.1422295	95	131
11	Petén & Gral. Emiliano Zapata, Sta Cruz Atoyac, 03320 Ciudad de México, CDMX	19.3665055, −99.1591011	92	199
12	Puebla 282 Roma Nte. 06700 Ciudad de México, CDMX	19.4211364, −99.1714281	92	216
13	Calle Salamanca 107, Roma Nte., 06700 Ciudad de México, CDMX	19.4172303, −99.1714257	91	139
14	Balsas 18 sineo, Miravalle 03580 Ciudad de México, CDMX	19.3605422, −99.1424208	88	215
15	Escocia & Calle Gabriel Mancera, Col del Valle Centro, 03100 Ciudad de México, CDMX	19.3876749, −99.1661223	87	220
16	Calz. de Tlalpan 2050, Campestre Churubusco, 04200 Ciudad de México, CDMX	19.3429739, −99.1434801	74	155
17	Calle Querétaro & Medellín, Roma Nte. 06700 Ciudad de México, CDMX	19.413905, −99.1672667	73	211
18	Av Sonora 149, Hipódromo, 06100 Ciudad de México, CDMX	19.4145946, −99.1714381	70	237
19	Calle Concepción Beistegui & Calle Yacatas, Narvarte Poniente 03020 Ciudad de México, CDMX	19.3873507, −99.1582722	69	178
20	Galicia Niños Héroes, Ciudad de México, CDMX	19.3886011, −99.1482661	69	111
21	Calle Enrique Rebsamen & La Morena Narvarte Poniente, 03020 Ciudad de México, CDMX	19.3985479, −99.1609147	61	97
22	Rancho Vista Hermosa & Rancho de Los Arcos, Parque Alameda del Sur 04929 Ciudad de México, CDMX	19.3069132, −99.124864	54	128
23	Bretaña & Irolo, Zacahuitzco, 03550 Ciudad de México, CDMX	19.3731238, −99.1398383	50	133
24	Gral. Emiliano Zapata 51 Portales Nte, Ciudad de México, CDMX	19.3642598, −99.1446719	47	131
25	Saratoga 714, Portales Sur, 03303 Ciudad de México, CDMX	19.3649279, −99.1540524	43	94
26	Sierravista & Calle Riobamba Lindavista Nte. 07300 Ciudad de México, CDMX	19.4940873, −99.1265294	41	117
27	Calle Salvador Díaz Mironn Sta María la Ribera Ciudad de México, CDMX	19.4492376, −99.1620973	40	85
28	Av. las Trancas 40 Narciso Mendoza 14390 U. Hab. Narciso Mendoza Super 6 Coapa, CDMX	19.292755, −99.125329	38	72
29	Calz. de la Viga 1756, Héroes de Churubusco, 09090 Ciudad de México, CDMX	19.3612758, −99.1240497	37	101
30	Avenida Santa Ana 300, Ex-Ejido de San Francisco Culhuacan, 04470 Ciudad de México, CDMX	19.3296075, −99.1272789	37	99
31	Coquimbo 07300 Ciudad de México, CDMX	19.4899307, −99.1281605	31	91
32	Calle Puente 222, San Bartolo el Chico, 14380 Ciudad de México, CDMX	19.2833487, −99.1373406	24	79
33	Paseo Galias 47 Lomas Estrella 2da Secc, 09890, Ciudad de México, CDMX	19.3205935, −99.0995659	23	56
34	Vicente Guerrero 40, San Gregorio Atlapulco, 16600, Ciudad de México, CDMX	19.2522187, −99.0614642	16	67
35	Av. México, San Gregorio Atlapulco, 16600 Ciudad de México, CDMX	19.2531664, −99.0513852,	15	74
36	Xochimilco-tulyehualco 191, Xochimilco, 16500, Ciudad de México, CDMX	19.2468579, −99.0835714	13	46
